# Supplementary material for: Identification and functional analysis of non-coding regulatory small RNA FenSr3 in Bacillus amyloliquefaciens LPB-18
Source: PeerJ. 2023 May 15;11:e15236. doi: 10.7717/peerj.15236 (PMC10194069; doi:10.7717/peerj.15236)
Supplement: Supplemental Information 4 [file peerj-11-15236-s004.zip › KO/CK-vs-T1_map/map04210.html]

KEGG PATHWAY: Apoptosis - Reference pathway


|  |  |
| --- | --- |
| **Apoptosis - Reference pathway** |  |

[
Pathway menu
| Organism menu
| Pathway entry
| Show description
| User data mapping
]

Apoptosis is a genetically programmed process for the elimination of damaged or redundant cells by activation of caspases (aspartate-specific cysteine proteases). The onset of apoptosis is controlled by numerous interrelating processes. The 'extrinsic' pathway involves stimulation of members of the tumor necrosis factor (TNF) receptor subfamily, such as TNFRI, CD95/Fas or TRAILR (death receptors), located at the cell surface, by their specific ligands, such as TNF-alpha, FasL or TRAIL, respectively. The 'intrinsic' pathway is activated mainly by non-receptor stimuli, such as DNA damage, ER stress, metabolic stress, UV radiation or growth-factor deprivation. The central event in the 'intrinsic' pathway is the mitochondrial outer membrane permeabilization (MOMP), which leads to the release of cytochrome c. These two pathways converge at the level of effector caspases, such as caspase-3 and caspase-7. The third major pathway is initiated by the constituents of cytotoxic granules (e.g. Perforin and Granzyme B) that are released by CTLs (cytotoxic T-cells) and NK (natural killer) cells. Granzyme B, similarly to the caspases, cleaves its substrates after aspartic acid residues, suggesting that this protease has the ability to activate members of the caspase family directly. It is the balance between the pro-apoptotic and anti-apoptotic signals that eventually determines whether cells will undergo apoptosis, survive or proliferate. TNF family of ligands activates anti-apoptotic or cell-survival signals as well as apoptotic signals. NGF and Interleukin-3 promotes the survival, proliferation and differentiation of neurons or hematopoietic cells, respectively. Withdrawal of these growth factors leads to cell death, as described above.

|  |  |  |
| --- | --- | --- |
| Reference pathway | 184% 150% 122% 100% 82% 67% 55% | 图片下载 |
